# Why is the muscle spindle so complicated?

**DOI:** 10.1113/EP093012

**Published:** 2025-09-12

**Authors:** Vaughan G. Macefield

**Affiliations:** ^1^ Department of Neuroscience, School of Translational Medicine Monash University Melbourne VIC Australia

**Keywords:** calcium channels, mechanotransduction, muscle spindle

1

The muscle spindle, one of my favourite sensory organs (along with the Ruffini ending, a much simpler receptor), is an encapsulated stretch receptor embedded within skeletal muscles. It is a complex structure that contains two types of mechanosensitive endings the primary (group Ia) and secondary (group II), and has been the subject of detailed physiological investigation for 100 years (since the first recordings from single afferents of muscle spindle primary endings by Adrian and Zotterman was published in 1926), yet its operation continues to yield surprises. As the only mechanoreceptor in the somatosensory nervous system with its own motor supply [the fusimotor (gamma) motoneurones, which regulate the sensitivity of the primary and secondary endings to stretch], it truly is a wonderful beast. But do we really know how it transduces stretch? Bob Banks and Guy Bewick have been erstwhile explorers hunting this beast and trying to uncover its secrets for several decades, and we now know much about the underlying physiology of the muscle spindle (Bewick & Banks, 2015), but the journey is far from over. Indeed, the muscle spindle is a gift that keeps on giving. Although it is known that PIEZO2 is required for the spindle ending to generate spontaneous activity and to respond to changes in muscle length (Woo et al., 2015), other factors are certainly at play.

For instance, did you know that synaptic‐like vesicles in the receptor ending release glutamate (the main excitatory neurotransmitter within the CNS) when stretch is applied and that this influences the sensitivity of the ending; moreover, did you also know that exogenously applied glutamate increases the firing of the afferents (Banks et al., 2002; Bewick et al., 2005)? Like many physiological processes, this autogenic facilitation by glutamate and the recycling of synaptic‐like vesicles depends on calcium, which is also essential for initiating the receptor potential. And now, perhaps, we know how. In this special issue, Simon et al. (2025) interrogate the different types of voltage‐gated calcium channels: in neurones, N and P/Q channels are expressed presynaptically and postsynaptically and subserve key roles in synaptic transmission, whereas the L‐type channels are found only on the cell bodies and proximal dendrites of neurones, postsynaptically. Using selective calcium channel toxins and pharmacological agents, the authors conclude that the N and P/Q channels are involved in regulating the stretch sensitivity of the spindle ending, whereas only the L‐type channel contributes to synaptic‐like vesicle recycling.

However, the story gets even more complicated. Not to be outdone, it now turns out that transient receptor potential vanilloid 4 (TRPV4), a non‐selective cation channel that features in diverse physiological processes and has been implicated in several neurological disease states (Zhang et al., 2025), is the new kid on the block for mechanotransduction. Simon et al. (2025) found that muscle spindle activity was completely blocked by selective TRPV4 antagonists and augmented by selective agonists, suggesting that this channel is essential for maintaining spindle afferent firing during muscle stretch. So, is the molecular basis of mechanotransduction in the muscle spindle really that complicated? You bet it is!

## AUTHOR CONTRIBUTIONS

Vaughan G. Macefield conceived of this work, wrote the manuscript and approved the final version of the manuscript. Vaughan G. Macefield agrees to be accountable for all aspects of the work in ensuring that questions related to the accuracy or integrity of any part of the work are appropriately investigated and resolved. All persons designated as authors qualify for authorship, and all those who qualify for authorship are listed.

## FUNDING INFORMATION

No funding was obtained.

## CONFLICT OF INTEREST

The author has no competing interests.
